# Scribble dictates orderly stem cell fate

**DOI:** 10.18632/oncotarget.4923

**Published:** 2015-07-21

**Authors:** Ryo Fujita, Daiki Seko, Yusuke Ono

**Affiliations:** Department of Stem Cell Biology, Atomic Bomb Disease Institute, Nagasaki University and Graduate School of Biomedical Sciences, Nagasaki, Japan

**Keywords:** Scrib, stem cells, skeletal muscle, satellite cells, polarity

Cell polarity has a variety of biological roles, including morphogenesis during development, the maintenance of tissue integrity, and adult tissue regeneration. In epithelial cells, cell polarity is generally regulated by three evolutionarily conserved protein complexes: the apical PAR and Crumbs complexes and the basolateral Scribble (Scrib) complex. Thus, dysfunction of these polarity proteins causes cell polarity defects and disorganization of tissue architecture, resulting in diseases including cancer. Accumulating evidence has also demonstrated that cell fate decisions in tissue stem cells are likely influenced by the polarized distribution of cell polarity proteins. These then act as potential determinants of asymmetric cell division, which allows a stem cell to generate a daughter cell that self-renews to maintain its own stem cell pool and another cell that undergoes differentiation, therefore ensuring a balance between self-renewal and differentiation [1].

Skeletal muscle tissue stem cells are satellite cells with remarkable regenerative capacity after muscle damage [2]. Satellite cells located between the basal lamina and surface of myofibers are normally quiescent but are swiftly activated in response to stimuli such as muscle injury. Activated cells then proliferate symmetrically and asymmetrically to generate both self-renewed cells that maintain the stem cell pool and myogenic progenitors that eventually undergo terminal differentiation to form new myofibers. Several factors that control asymmetric cell division of satellite cells have been described: the Notch antagonist Numb is located in committed myogenic progenitors during cell division [3]; unequally distributed polarity proteins PAR3 and aPKC asymmetrically activate the p38 MAPK pathway to promote the transcription of a myogenic regulatory factor MyoD, which promotes cells to undergo myogenic differentiation [4, 5].

A recent study published in *Cell Reports* further demonstrated that the cell polarity protein Scrib is a crucial regulator of satellite cell fate decision [6]. Activated satellite cells up-regulate Scrib, which is then asymmetrically distributed in dividing cells. Scrib protein accumulates in high quantities in daughter cells committed to myogenic differentiation and in low quantities in proliferating or self-renewing cells. Because Scrib is a tumor suppressor that possesses potent anti-proliferative activity in epithelial cells, satellite cells were expected to show increased proliferation when levels of Scrib were reduced. Interestingly, however, satellite cells lacking *Scrib* failed to expand their progeny. Indeed, satellite cell-specific *Scrib*-null mice exhibited a severe regeneration defect with a significant reduction of myogenic progenitors after cardiotoxin-induced muscle injury.

Given that *Scrib*-inactivated satellite cells lost their proliferative ability, the study next focused on how proliferation was affected by the loss of Scrib [6]. The genetic ablation of *Scrib* resulted in a decreased expression of phosphorylated c-Jun that sustains the proliferative state of satellite cells. Moreover, *Scrib-*deficient cells were less responsive to stimulation by growth factors such as IGF-1. Indeed, Scrib appears to act as a signaling scaffold to modulate growth factor signaling pathways for the maintenance of myogenic progenitor expansion. However, in contrast, the overexpression of Scrib suppressed cell proliferation and promoted myogenic differentiation. It is also of interest to note that satellite cell self-renewal was impaired by the retrovirus-mediated constitutive expression of *SCRIB*, indicating that the down-regulation of Scrib was a necessary step for the self-renewal of satellite cells. Taken together, these results suggest that satellite cell fate decisions determined by Scrib are dose-dependent, and thus, an appropriate level of Scrib might be crucial for the balance between population expansion, differentiation, and self-renewal in satellite cells.

Considering the evidence that Scrib is a key cell polarity protein that prevents the outgrowth of tumor cells in epithelial tissues, Scrib is unlikely to function as a universal anti-proliferative factor, and thus its role is likely to be varied and depend specifically upon its cell and tissue distribution. It remains unclear whether the function of Scrib is associated with other polarity regulators such as PAR for the fine-tuning of satellite cell fate. Further studies using genetically engineered mouse models are needed to uncover how polarity proteins regulate the molecular events involved in cell fate determination. This will hopefully support the development of stem cell-based regenerative medicine for muscle wasting diseases such as muscular dystrophies and age-related sarcopenia, as well as for cancer biology.
